# Collectanea

**Published:** 1830-06

**Authors:** 


### COLLECTANEA.

Floriferis ut apes in saltibus omnia libant,

Omnia nos, itidem, depascimur aurea dicta.

### PATHOLOGY.

*Gonorrhœa and Syphilis.* The common notion prevails, that proper gonorrhœa, being a simple inflammatory secretion of the mucous membrane, and not acting as a poison upon the system, the lues or syphilis is altogether a distinct disease. Let us examine this opinion. I have said, that whilst on both sides the surfaces remain sound, no constitutional disease is discoverable. But before we decide that the matter secreted in gonorrhœa and in syphilis have no relation to each other, we must shew that the gonorrhœal secretion being absorbed into the circulation, as in the case of superficial sores, is incapable of producing constitutional, or as they are called secondary, symptoms. Now I aver that such a connecting link between these diseases exists, and is palpable to observation. I have already described the symptoms arising from the absorption of the gonorrhœal matter, and it cannot, I think, be denied that the resemblance is such to those of syphilis, as to established their very intimate relation, if not their identity. If a constitutional disease be traceable through the medium of gonorrhœal sores in a subject hitherto immaculate, the next step in the inquiry is to determine the operation of a constitution already tainted with the poison of gonorrhœa upon sores of this description. Are not the natural secretions of the bowels, the skin, and the kidney, influenced by the deranged state of the constitution? Are not the morbid discharges from simple wounds and ulcers, having their origin in casualties, also subject to vitiation from a similar influence? We know that the unhealthiness of the matter of ulcers and suppurating surfaces, of whatever description, is constantly and truly referred to a prevailing morbid state of the system. Thus, if a poisoned habit contracts a sore, though the sore may have been caused by an accidental læsion of the skin, it becomes at once contaminated, and secretes a virus possessing properties not observed to belong to the secretion of a fresh and healthy system: the property, for example, of exciting upon a new surface an inflamed vesicle or pustule, which is followed by an excavated ulcer, instead of a superficial, raised, or level sore, and which runs into phagedenic ulceration, whether on the glans penis or the tonsil; of affecting parts of the system not within the ordinary range of the milder poison, as the iris and the periosteal membrane; of exhibiting cutaneous eruptions, peculiar in colour, figure, &c., and differing somewhat in other respects, though that remains for future search to determine, from the class to which they respectively approximate.

From this observation it would seem that the gonorrhœal and syphilitic poisons are the same in kind, and that the only difference between them consists in the degree of their intensity and the extent of their operation. The purely *local* production of the gonorrhœal matter, prior to the participation of the constitution, would lead us to expect a wide difference between the secondary symptoms of gonorrhœal sore and those of lues; in which last the sore is as much constitutional as local, and the matter secreted is in effect a poison. For I consider that the primary sore of lues is in its nature a constitutional or secondary sore, having originally been the production of a system already impregnated with the venereal poison. The matter of poison being once engendered, its communication to another requires no such conditions as a breach of surface or an impure constitution in the recipient. In its most intense and concentrated form it may at once be communicated by its proper irritation and inflammation to a novice, and the recurrence of the circumstances stated as explanatory of its origin and derivation from the gonorrhœal stock, must be sufficiently frequent to perpetuate and renovate the matter of infection without limit.

It appears that a simple or primary gonorrhœal sore may, and frequently does, communicate a constitutional disease, bearing incontestable evidence of a poison analogous in general character, but milder in degree, more limited in the sphere of its operation, and from this and other circumstances capable for the most part of being distinguished from that of syphilis. The signs of distinction are becoming artificial and obscure, and the bases of them will in all probability be eventually overlooked and forgotten. Further, a sore of any kind formed on the genitals of a person whose blood circulates the gonorrhœal poison, becomes capable by its secretion *sui generis*, the type of the poison, of communicating syphilis, viz. of raising a vesicle or pustule, followed by a circumscribed, excavated, hard-edged ulcer, which, if not restrained by the action of mercury, is disposed to extend in depth and breath, and, in short, destroy substance indefinitely, which is the local characteristic of the most active syphilitic poison.

From what has been stated I derive the following conclusions:

1. That absorption does not take place from sound surfaces, and therefore the poison of gonorrhœa, if it be one, is not developed in the system. In the very rare cases in which constitutional symptoms follow gonorrhœa in the absence of a visible sore, I refer their existence to absorption from an ulcer in the urethra.

2. The gonorrhœal matter, though apparently the simple secretion of an inflamed surface, is capable, when absorbed into the system, as from sores, of acting as a poison in the production of constitutional symptoms.

3. That the venereal poison is essentially one; for analogous secondary or constitutional symptoms succeed to analogous primary sores, in systems previously healthy.

4. That the distinction between the gonorrhœal and syphilitic orders of symptoms, primary or secondary, is demonstrative of the difference between the secretions of a system previously healthy and the secretions of a system already charged with a poison.—*Observations on the Pathology of Venereal Affections, by* BENJAMIN TRAVERS, F.R.S. &c.

---

*Cases of simple Ileus.* (From Dr. ABERCROMBIE *on Diseases of the Stomach.*)

*Ileus fatal in the State of Distention, without Inflammation.* A man, aged forty, 20th August, 1814, had violent pain of the abdomen, urgent vomiting, and costiveness. The pain was at times increased by pressure, but not uniformly so; his pulse was generally about 96, but at last rose to 120. The attack had commenced with symptoms resembling cholera, which had speedily passed into those of ileus. Repeated bloodletting and the other usual means were actively employed, and his bowels were moved on the 29th, but without relief. I saw him on the 30th. His abdomen was then distended, tense, and tympanitic; his strength was rapidly sinking; and he died the same afternoon. For some time before this attack, he had been affected with slight symptoms, which had been referred to the liver.

*Inspection.* A large portion of the small intestine was in a state of great and uniform distention, without any appearance of inflammation. The lower part of the right lobe of the liver was unusually soft. No other morbid appearance could be discovered on the most careful examination.

In the symptoms of this case at its commencement, there was a complication which, perhaps, may remove it in some degree from the correct history of ileus; though the fact of cholera passing into ileus is by no means uncommon, and the fatal symptoms were simply those of ileus. The following, perhaps, was a more decided example, and showed the affected parts in the state of high distention, with a slight and recent blush of redness, not amounting to inflammation, or, at least, not to such a state of it as could be considered the fatal disease.

A woman, aged twenty, 23d June, 1813, was affected with violent pain at the upper part of the abdomen, extending towards the left side, and at times increased by pressure; frequent and violent vomiting, and obstinate costiveness. The belly was distended and tense; the tongue white; pulse 76, and small. On the 16th, she had got wet during the flow of the catamenia, which ceased, but returned at night; pain about the umbilicus began on the 17th, and increased gradually; vomiting began on the 21st, with hiccup. Blood-letting, with various purgatives, injections, warm bath, &c. were actively employed by a physician of eminence.

24th.—Incessant screaming from the violence of pain; frequent hiccup; no stool; pulse 88, and small; frequent vomiting; belly distended and tender; every medicine was instantly vomited.

25th.—No stool; every thing vomited; pain almost gone; pulse very feeble.

26th.—No stool; free from pain; vomiting continued, with hiccup. Died in the night.

*Inspection.* The whole of the colon, and about twelve inches of the lower extremity of the ileum were empty, contracted, of a white colour, and seemed perfectly healthy. The remainder of the small intestine was distended to the greatest degree, so as to appear thin and transparent; its contents were chiefly watery matter and air. On the surface of the distended intestine, there was on several places, especially at the lower part near the contracted portion, a superficial blush of vivid redness, but without any appearance of exudation. There was a small abscess in the left ovarium. All the other parts were healthy.

A remarkable feature in this case is the mode of its termination, namely, by rapid sinking and cessation of pain, resembling the symptoms of internal gangrene, yet with the inflammatory appearance in its earliest stage. It is also to be observed, that the pain was increased by pressure as early as the 23d, when we can scarcely suppose any inflammation to have existed; and the same happened in the former case, where there was no appearance of inflammation.

*Ileus fatal with Distention, and a dark Livid Colour of the Parts without Disorganization.* A lady, aged seventy, after her bowels had been confined for several days, was seized on the 5th January, 1820, with violent pain of the abdomen and vomiting; pulse natural. The usual means were employed by Mr. White without relief. On the 6th, the pain was considerably abated, but there was severe sickness, with frequent vomiting, and obstinate costiveness; the pulse from eighty to ninety. The belly was natural to the feel, and without any degree of tenderness. On the 7th, the same symptoms continued; the pulse eighty. Towards the afternoon, sinking began to take place, and she died in the night.

*Inspection.* The colon contained a great deal of hardened feces, but appeared quite healthy and without any flatulent distention. The lower extremity of the ileum, to the extent of eighteen inches, was empty, contracted, and of a white colour, like the intestine of an infant; immediately above this, a portion from eighteen to twenty-four inches in extent was throughout of a livid brown colour, or nearly black, but without disorganization or softening, and without any appearance of exudation. This portion was considerably distended, and the whole of the remaining part of the small intestine to the very commencement of the canal was in a state of uniform and great distention, and of a dull leaden colour, with here and there portions of a dark livid brown. It continued only thin fluid feces and air. There was considerable disease of the internal surface of the abdominal aorta. The other parts were healthy.

The part chiefly affected in this case would appear to have been in an intermediate stage of that condition which passes into gangrene; and, it is worthy of observation, that it was without any appearance of inflammatory exudation.

*Ileus fatal by Gangrene without Exudation.* A boy, aged twelve, (26th Oct. 1813,) was affected with violent pain of the belly, chiefly round the umbilicus, urgent vomiting, and costiveness for two days; abdomen distended, pulse fifty. Various remedies were employed without benefit. On the 27th, the pulse rose to 120, with increase of the pain, tension and tenderness of the abdomen. Bloodletting was used in the morning, and again at three P. M.; after which the pulse fell to 112. The other usual means were employed without procuring any evacuation from her bowels; the pain continued unabated; sinking took place, with coldness of the body; and he died between seven and eight o'clock in the evening, having continued in violent pain until immediately before death. I did not see this case during the life of the patient, but was present at the examination of the body.

*Inspection.* The stomach was healthy; the small intestine was a little distended and slightly inflamed; especially at the lower part, where it had contracted some adhesions. The whole right side of the colon was in a state of gangrene, especially the caput cœcum, which had burst and discharged into the cavity of the peritoneum a large quantity of fluid feces. The diseased parts appeared to have been much distended, and, after being emptied by the rupture, had not contracted, but had fallen flat, presenting a very broad surface like an empty bag. There was no inflammatory exudation; and, at the upper part of the ascending colon, this diseased part terminated at once in healthy intestine, which was white, collapsed and empty. This was the state of the remainder of the colon, except the sigmoid flexure, which, with the rectum, contained much consistent feces.

*Ileus fatal by Gangrene combined with Exudation.* A young man, aged nineteen, (17th Oct. 1813,) was affected with violent pain round the umbilicus; incessant vomiting; abdomen hard, tense, and tumid; bowels obstinately costive; pulse 84; countenance depressed and anxious. He had been ill six days, during which a variety of remedies had been employed without relief. He was now treated by repeated general and topical bleeding, blistering, various purgatives, purgative and tobacco injections, and all the other usual remedies, but without any permanent relief. On the 18th, the pulse was 120, and the belly tympanitic; the vomiting was urgent, but not feculent, and there was some slight feculent discharge by the injections. On the 19th, the symptoms were somewhat abated; but on the 20th, they again increased; the pain violent, the vomiting incessant, the belly much distended: the pulse from ninety-two to ninety-six; slight discharge of watery matter by stool. He died on the 21st.

*Inspection.* The stomach was healthy. Almost immediately below it, the intestine was distended to the greatest degree. It was in some places thin and transparent; in others, highly inflamed and gangrenous, and bursting when handled; and in others firm, though perfectly black. This state continued to the middle of the small intestine, where a portion, twelve inches in length, was empty, contracted, and healthy. Below this, the canal was again diseased as in the parts above, distended, inflamed, gangrenous, and adhering by extensive exudation, until three inches from the extremity of the ileum, where it became again contracted, empty, and of a healthy colour. These contracted portions were quite pervious, easily dilated, and, in their coats, appeared perfectly healthy. The colon was healthy and collapsed, except at its lower part, where it contained some consistent feces. The distended portions of intestine were chiefly filled by air; there was in some places thin feculent matter, but in small quantity; and no consistent feces could be found in any part of it.

---

### PRACTICAL MEDICINE.

*Ergot in Intermittent Fever.* It is stated in Rust's Magazine, vol. xxix. No. 3, that Dr. MEHLHAUSEN, of Eilau, has employed the ergot in intermittent fever, and he is persuaded that it is a remedy deserving the attention of physicians in that disease. In the autumn of 1828, he made his first trial with the article, by prescribing it in seven uncomplicated cases, five of which were entirely cured by it, viz. three quartans and two tertians: one of the remaining cases was removed from under his care soon after the treatment was commenced; in the other, a debilitated subject, sixty years of age, the ergot produced no effect, and the cure was effected by the bark. The ergot was given in doses of ten grains every two hours before the expected paroxysm. In debilitated persons, only six to eight grains were given at once. Of course, this remedy is totally inadmissible where there is the least suspicion of pregnancy.

---

*Sulphate of Copper as a Remedy in Croup.* In Hufeland's Journal for May 1829, Dr. FIELITZ relates five cases of croup, for the purpose of proving the efficacy of sulphate of copper as a remedy in that disease. We give the following as examples.

CASE I. Emily S—, three years old, of a weakly constitution, and inclined to scrofula, had lost an elder sister five days before by the croup, and was attacked, January 23, 1824, with fever, a short hoarse cough, indistinct articulation, and laborious respiration, with some degree of rattling in the chest.

An emetic was directed, and followed in the evening by four grains of calomel. A considerable quantity of viscid slime was discharged by vomiting. In the evening, all the symptoms augmented, the cough assuming more and more the croupy sound. By the emetic the bowels had been once opened. Four leeches were now applied upon the neck, and a blister over the upper part of the sternum; every two hours a quarter of a grain of sulphate of copper in sugar being administered. By the first doses vomiting was in a few minutes produced, by which a quantity of viscid mucus was discharged. After midnight the child became more composed, the skin was covered for several hours with moisture, and towards morning a tolerably quiet sleep ensued. The next morning all the symptoms were found to be greatly abated. An eighth of a grain of digitalis was added to each dose of the sulphate of copper, and for drink lukewarm sugared water. During the day the cough and respiration lost entirely the croupy sound, and several hours of sleep were obtained at night; interrupted, however, by occasional attacks of a loose cough. By the third day the patient became sprightly, cheerful, and very shortly entirely free from all disease.

Case II. Julius R., three years old, stout and healthy, was, on the 28th of February, 1824, suddenly taken ill with a severe hollow cough, indistinct articulation, very difficult respiration, attended by symptoms of suffocation. The slightest pressure upon the larynx caused the patient to scream aloud. The face was dark red and tumid, the eyes glassy, mouth and tongue parched, the whole body hot and dry; pulse quick, hard, and contracted; violent pulsation of the arteries of the neck; urine passed involuntarily. Five leeches were directed to the throat, the bleeding from which to be kept up for three hours, followed by a blister on the upper part of the sternum, and every two hours a quarter of a grain of sulphate of copper in sugar.

Next day, patient better; had passed a tolerable night. The first doses of the Sulph. Cupri had occasioned repeated vomiting. Cough and hoarseness still continue. At noon, the patient was covered with a profuse sweat; had slept; cough and respiration more free. In the evening, exacerbation of fever, with increase of the other symptoms. An eighth of a grain of Digitalis was added to each dose of the Sulph. Cupri.

March 1st.—During the whole night, patient in a profuse perspiration; the cough has lost its croupy sound, other symptoms abated. An emetic of twelve grains of Sulph. Cupri had produced a discharge of a quantity of very consistent mucus. The powders to be discontinued until evening, and a mixture of the muriate of Ammonia given.

By the 4th, all the symptoms of the disease had disappeared.

On the 12th, the child having ventured out too soon, the disease returned, but with less violence. Two grains of Sulph. Cupri were administered, and produced frequent vomiting of a thick mucus. Minute doses of the Sulphate of Copper and Digitalis were then commenced with, by which, and light diaphoretics, in a week the patient was entirely cured.

---

### SURGERY.

*Prolapsus Ani, treated after the manner of* Mr. Hey. By Dr. Macfarlane. —*Glasgow Med. Journal.*

W. A., shoemaker, æt. fifty-four, became a district patient in February. The gut descended for more than two inches in every attempt to pass the feces, accompanied with great pain and tenesmus. When he remained for a few minutes in an erect position, the same displacement took place slowly, although no propulsive efforts were made: this, however, could be prevented by pressure. The first part projected from the anus was a circular fold of the mucous membrane of the rectum, at its verge, of a livid colour and tuberculated appearance; and this was soon followed by the complete descent of the bowel, and hemorrhage from many points. The recumbent posture, and gentle but continued pressure for a few minutes, generally effected the reduction of the prolapsus, although at an earlier period it often continued irreducible for hours. The general health of the patient was much impaired, and he was prevented from following his employment by the constant irritation and almost daily attacks of hemorrhage.

On examining the anus after the gut was replaced, the surrounding integuments were found extremely relaxed. There existed such an unnatural looseness in the attachment of the skin around the anus to its corresponding cellular membrane, that it could be easily drawn out with the fingers in the form of one or more large flaps.

Having succeeded, in two similar cases which came under my care in the Royal Infirmary, in completely curing the disease by cutting off the loose integuments, as recommended by the late Mr. Hey,* I determined to try it in this case. The skin was drawn out as far as possible into broad flaps, and cut off with the scissors in a circular direction, until all the superfluous integument was removed, including a portion of the livid and tuberculated fold of the mucous membrane, which was projected from within the sphincter. The pain was trifling, and only a few drops of blood were lost. A soft compress and a T bandage were applied, and he was strictly confined to bed. For a few days a partial procidentia took place on every attempt to go to stool. He had a good deal of pain and inflammation around the anus, attended with complete retention of urine, which required the frequent introduction of the catheter. In ten days after the operation, he was able to walk about and void his stools, without any return of the disease; and in three weeks he was perfectly cured. Pressure was continued to the part for some time longer; occasional doses of castor oil were prescribed, and he was enjoined to avoid straining at stool.

There will generally be found, in obstinate and long-continued forms of this disease, a great relaxation in the connexion of the rectum at its lower part with the surrounding textures. This circumstance, although it may not be the original cause, is sufficient in many cases to account for the displacement in chronic and inveterate cases, although I believe it is generally accompanied by a diminished power of the sphincter. If the rectum, in consequence of being much irritated, as in various bowel complaints, ultimately becomes relaxed, the tenesmus, which is an invariable attendant, may so overcome the sphincter as to give rise to a procidentia: but when, as in the case now detailed, an erect position is sufficient to cause the descent of the gut, we have grounds for believing that, besides the relaxed state of the rectum, there exists a want of power in the sphincter muscle, which part, along with the levator ani, is mainly instrumental in maintaining the rectum in its natural situation. In the cases detailed by Mr. Hey, there existed, in combination with relaxation of the integuments, one or more livid tubercles at the verge

* Practical Observations in Surgery, 2d Edit. p. 444.

of the anus, which were also removed. He expected from this operation that inflammation of the surrounding cellular texture would be excited, the attachments of the rectum consolidated, and the power of the sphincter improved. In a majority of cases, the disease will be found to yield (although the cure is often tedious and protracted) to the local applications and internal remedies usually employed. Should it continue, however, as sometimes happens, after the exciting cause has been removed, we shall occasionally find that the loose state of the skin around the anus and the relaxed attachments of the rectum at its termination, become the primary causes of the continuation of the disease. It is, I conceive, in such circumstances that this simple operation may be beneficially adopted.

---

*Case of Umbilical Hernia treated by Ligature.* By Mr. Robert Cowan, Surgeon. (*Glasgow Med. Journal.*)

In 1827, I was called to visit a child about three months old, which, on examination, was found to have an umbilical hernia, as large as an hen's egg. No herniary tumor existed at birth, nor for at least ten days afterwards; and the tumor had been noticed only the day before I saw it, by the child's mother, after the nurse (who in all probability conceived it to be owing to her carelessness) had left the house. The child had experienced repeated attacks of colic, attended with such an irritable state of the bowels, as to require opiate clysters and opiates to be frequently administered.

Attempts were made, by means of a compress and bandage, to keep the hernia reduced, and allow the aperture through which it had passed to become obliterated; but these proving ineffectual, the plan of applying half of a ball sufficient to cover the opening, and securing it by means of adhesive plaster, as recommended by Sir Astley Cooper, was tried, and with equal want of success. These attempts proving abortive, and the frequent renewal of the bandages being a great source of annoyance to the infant, it was resolved, notwithstanding the arguments advanced by Scarpa and others against the employment of the ligature, to attempt a cure by its means, in the mode adopted by Desault.

Accordingly, the child having had the previous day a dose of castor oil, with the assistance of my friend Dr. John Cooper, the hernia, which was entirely intestinal, was reduced, and a small round ligature firmly applied around the neck of the sac, as close to the abdomen as possible. This gave little uneasiness; and a warm bath, prescribed in the evening, procured a comfortable night. On the third day a fresh ligature was applied, which produced sloughing of the part: on the fifth a third ligature was applied; and on the seventh day from the operation the included portion dropped off, leaving a small sore, which was dressed with dry caddice, a small compress, and a strip of adhesive plaster.

In another week the sore was entirely healed, but the compress and strap were continued for three weeks longer, at which time the aperture in the umbilicus was as completely and as firmly closed as in children for the same age.

I have since occasionally examined the part, and the cure is complete.

In the umbilical hernia of children, the protruded parts escape directly through the umbilical ring; in that of adults, unless the hernia has existed from infancy, the aperture is in the abdominal parietes near the umbilicus, never in the umbilical ring itself.

What distinguishes the umbilical hernia of childhood from every other species of hernia, is the strong natural tendency to contract, which exists till about the age of nine years, in the aperture through which the viscera protrude ; and which contractile tendency, greatest immediately after birth, gradually decreases till about the period mentioned, when it ceases altogether.

In some instances umbilical hernia is congenital, and when so, is not unusually accompanied with malformation of the abdominal muscles. Most frequently, however, it first commences about the age of two or three months*, and unless attended to is apt to increase in size, and prove a continual source of irritation and annoyance. The inherent tendency in the aperture through which the viscera pass to contract, is in many cases strong enough to produce a natural cure, but more generally the aid of art is requisite.

Two plans of cure have been proposed, viz. compression and the ligature. By means of the former, the protruded viscera are reduced and the aperture kept closed, either by bandage and compress, or by introducing a segment of a small ball into the opening. It is quite obvious that this latter mode is objectionable, from the ball producing precisely the same kind of resistance to the closure of the aperture as the viscera did. The plan of cure by compression is most generally adopted.

The other method of cure is by the ligature, as in the preceding case ; and this may either be employed when compression has failed, or, as was the practice of Desault, in preference to it.

Celsus, in his Chapter de Umbilici Vitiis, describes the mode of curing umbilical hernia by the ligature. He, however, makes no distinction between umbilical and ventral herniæ ; and so far from being aware that this operation was likely to be most successful with infants, he expressly forbids its performance on any below the age of seven, or above that of fourteen years.† The operation by the ligature seems not to have been practised in modern times till Saviard operated, and that successfully, on two children under fourteen months old. His example was not followed, and till the time of Desault was entirely neglected.

Desault revived the use of the ligature in the umbilical hernia of children, an operation founded on a correct idea of the anatomical structure of the part ; and especially on the fact that the opening through which the viscera pass has the strong natural contractile tendency already alluded to. It has been alleged that Desault revived the operation described by Celsus ; but the absurdity of this idea is rendered apparent by the quotation already made from that author's works. Desault operated on at least fifty patients, none of whom he lost ; and from the result of these he has drawn the following conclusions : 1st, That the sooner after birth the operation is performed the more certainly will a cure be effected ; 2dly, That at the age of four years a cure is effected with some degree of difficulty ; and 3dly, That, at nine, the contractile tendency in the aperture being almost entirely lost, the cure is nearly impossible.

The operation for the cure of umbilical hernia by the ligature has, since the

* Desault.

† Nam curationi neque infans, neque aut robustus annis aut senex aptus est sed a septimo fere anno ad quartum decimum.—*Celsus de Medicina*, vol. ii. p. 327.

death of Desault, fallen into disuse; and to this the opinion of Scarpa has, in no small degree, contributed.

It has been objected to this operation, that it is sometimes ineffectual. This is certainly the case if performed when the contractile disposition in the ring has ceased; and the same objection may with equal propriety be asserted of the mode by compression.

Others allege it to be dangerous, especially if a portion of intestine is included in the ligature. When properly and carefully performed, this objection can never occur. Inflammation, irritation, and even death, it is said, have followed the operation, even when every thing has been well done. This is very possible, and yet in Desault's fifty cases no death took place.

When the ligature has succeeded at first, it is alleged the cicatrix has afterwards given way, and a hernia formed a second time.

I confess it was with surprise I found, in the work of Scarpa, this opinion attempted to be supported by a supposed analogy between the operation of the ligature in the umbilical hernia of children, and the barbarous treatment of inguinal hernia by the ligature of the hernial sac and spermatic cord in the adult. It is stated that, in this latter case, the cicatrix is apt to give way unless a truss is continually worn.

The two cases bear no analogy to each other: in the inguinal hernia the opening possesses no disposition to contract, while that in the umbilical hernia of children does so: and, besides, when a cure has followed the use of the ligature in umbilical hernia, the navel is at least as firm as in those children who never have had this disease.

M. Cartier also, overlooking the anatomical structure of the navel, declares, that since "la cicatrice n'empéche pas toujours la recidive de la hernie inguinale ou crurale chez les sujets qui en ont été operés, on ne doit pas, à plus forte raison, compter sur une guerison radicale aprês l'opération de la hernie de l'umbilic chez les enfans."

This opinion is not corroborated by experience; for, as stated above, the navel is as strong after the termination of the cure of the umbilical hernia by the ligature, as it is in those infants who have never had hernia.

Dupuytren has operated on umbilical hernia in children by the ligature many times, and always with the happiest effects; and many cases are scattered over the pages of our numerous periodicals in which the operation met with equal success.

In the umbilical hernia of children, I would, in deference to the expressed opinion of the profession, first attempt the cure by compression merely; if this failed, or was very annoying to the child, from the difficulty of retaining the necessary bandages *in situ*, recourse would be had to the ligature, as in the preceding case. From the experience of Desault, as recorded by Bichât, it is fair to infer that, under proper limitations, this operation is both safe and efficacious; and though, in the greater number of instances, a cure is possible by compression alone, still there are others where this fails, and in which the ligature will succeed.

---

*Chlorate of Lime successfully employed in a Case of Cancer Aquaticus.* By M. BERNDT. (*Journ. der Pract. Heilkunde.*)

The Noma, or aquatic cancer, is a species of gangrenous pustule of the lips, peculiar to children, and of the nature of which no definite opinions have yet been formed. The local application of the pyroligneous acid has been much vaunted in this disease. In the present case it failed, as well as the hydrochloric acid, and many internal remedies. Mr. B. then applied the chlorate of lime to the ulcer, and in the course of a few days the wound assumed a favorable aspect, and it soon healed. The chlorate of lime was made into a paste with a small quantity of water, and applied to the lip of the child. It was applied every two hours during the day, and three times in the night. In proportion as the severity of the disease was diminished, the remedy was less frequently used, and at the end of eight days it was given up altogether, and an ointment substituted of balsam of Peru and myrrh.

